# Estimation of the rate and number of underreported deliberate self-poisoning attempts in western Iran in 2015

**DOI:** 10.4178/epih.e2017023

**Published:** 2017-06-15

**Authors:** Mehdi Moradinazar, Farid Najafi, Mohammad Reza Baneshi, Ali Akbar Haghdoost

**Affiliations:** 1 Modeling in Health Research Center, Institute for Future Studies in Health, Kerman University of Medical Sciences, Kerman, Iran; 2 Research Center for Environmental Determinants of Health (RCEDH), Kermanshah University of Medical Sciences, Kermanshah, Iran

**Keywords:** Suicide, Attempted, Underreporting, Poisoning, Iran

## Abstract

**OBJECTIVES:**

Rates of attempted deliberate self-poisoning (DSP) are subject to undercounting, underreporting, and denial of the suicide attempt. In this study, we estimated the rate of underreported DSP, which is the most common method of attempted suicide in Iran.

**METHODS:**

We estimated the rate and number of unaccounted individuals who attempted DSP in western Iran in 2015 using a truncated count model. In this method, the number of people who attempted DSP but were not referred to any health care centers, n_0_, was calculated through integrating hospital and forensic data. The crude and age-adjusted rates of attempted DSP were estimated directly using the average population size of the city of Kermanshah and the World Health Organization (WHO) world standard population with and without accounting for underreporting. The Monte Carlo method was used to determine the confidence level.

**RESULTS:**

The recorded number of people who attempted DSP was estimated by different methods to be in the range of 46.6 to 53.2% of the actual number of individuals who attempted DSP. The rate of underreported cases was higher among women than men and decreased as age increased. The rate of underreported cases decreased as the potency and intensity of toxic factors increased. The highest underreporting rates of 69.9, 51.2, and 21.5% were observed when oil and detergents (International Classification of Diseases, 10th revision [ICD-10] code: X66), medications (ICD-10 code: X60-X64), and agricultural toxins (ICD-10 codes: X68, X69) were used for poisoning, respectively. Crude rates, with and without accounting for underreporting, were estimated by the mixture method as 167.5 per 100,000 persons and 331.7 per 100,000 persons, respectively, which decreased to 129.8 per 100,000 persons and 253.1 per 100,000 persons after adjusting for age on the basis of the WHO world standard population.

**CONCLUSIONS:**

Nearly half of individuals who attempted DSP were not referred to a hospital for treatment or denied the suicide attempt for political or sociocultural reasons. Individuals with no access to counseling services are at a higher risk for repeated suicide attempts and fatal suicides.

## INTRODUCTION

Treatment for attempted suicide is an important social and health problem in many countries, accounting for a great part of primary and secondary health care [1]. Deliberate self-poisoning (DSP) is the most common method of suicide around the world [2] and accounts for 80% of suicide attempts (SA) globally. While DSP-based SA result in fewer deaths than other methods [3,4], the rate and risk of repetition of DSP is very high [4,5].

The most important solution for reducing the rate of SA and the mortality caused by SA is to identify and help those who have attempted suicide with counseling services [6]. Studies conducted on SA, particularly by poisoning, have regrettably shown that the number of attempted suicides is subject to undercounting, underreporting, and denial due to political, cultural, and social reasons, as well as the taboo among individuals and societies at large. The more influential these issues, the more prevalent the underreporting and undercounting of SA [4].

Levels of undercounting, underreporting, and denial of SA are higher for methods that are virulent and fatal rather than violent and aggressive. The majority of individuals who attempt suicide risk repeating it if they are not identified and do not receive health care or counseling. In addition, identifying the frequency of SA can help health care providers plan better programs and provide proper services [2].

Advanced statistics have provided different methods to estimate the rate of undercounting and the size of hidden populations, including the number of people who have attempted suicide [7]. One of these methods is the truncated count model, the advantages of which include precise estimation, simplicity, and the use of available data [8]. These attributes have prompted the extensive use of this method in areas of public health and epidemiology to estimate the size of populations such as criminals [9,10], drug abusers [11-14], and homosexuals [15].

Given the aforementioned reasons and the lack of accurately reported information on the number of attempted suicides, the aim of this study was to estimate the rate of DSP attempts in western Iran (the city of Kermanshah).

## MATERIALS AND METHODS

### Data sources

The study population consisted of all individuals in the city of Kermanshah who attempted suicide by DSP (International Classification of Diseases, 10th revision [ICD-10] codes: X60-X69) in 2015 and were taken to the Imam Khomeini Hospital, the principal poisoning treatment center in the west of the country. Kermanshah is the westernmost city in Iran with a population of 1 million people, and it shares a common border with Iraq. The poisoning center at the Imam Khomeini Hospital is the largest and only center for poisoning in western Iran. More than 90% of nondeliberate and deliberate poisoning cases are referred to this center, regardless of severity. The patients in our study who had attempted suicide were not discharged until they had physically and mentally recovered for 8 hours. After their condition had stabilized, information on the reasons for attempting DSP was collected by psychologists through routine interviews in a quiet, peaceful, and secure environment. Information was gathered on demographic variables; causes of SA; history of SA among family members and friends; time of incidence; poisoning agent; psychological conditions; and drug abuse of substances such as alcohol, heroin, opium, and morphine. Information was first gathered on paper and then recorded electronically.

In order to determine the number of attempted suicides, forensic data that were gathered daily and reported monthly by forensics employees who interviewed family members of the patients were used in addition to the hospital data (such as treatment documents and records). By integrating data based on national identification codes, all records of individuals who had attempted suicide were extracted and the number of attempted suicides was determined for each person.

### Truncated count estimators

Data on DSP can be used to estimate the number of attempted suicides (≥ 1 time). However, these data cannot be used to estimate n_0_, which is the number of individuals who have attempted DSP but were not referred to the poisoning center for treatment. The total number of individuals who have attempted DSP can more accurately be estimated by determining the value of n_0_ and adding it to the number of observed DSPs (N_total_= n_observed_ + n_0_). There are several estimators for estimating the population size of number total with truncated count data. The most important estimators include the Chao, Zelterman and mixture estimators. The population size according to the Chao estimator is N= n_1_^2^/(2n_2_)+n, where n_1_, n_2_, …, n_m_ refer to the number of observed attempted suicides (n_observed_). The population size according to the Zelterman estimator is N= n/1–exp(–2n_2_/n_1_).

In the mixture method, problems of heterogeneity that are caused by grouping homogeneous observations into several strata disappear. In this method, there is a mixture of *k* components, where each component follows a certain parametric distribution. The mixture method includes negative binomial, zero-inflated Poisson, and zero-inflated negative binomial models that cater to different dispersion parameters and heterogeneity aspects. Therefore, the mixture method often deals with additional dispersion problems. In the truncated count model, mixtures of truncated count data give a lower mean square error (MSE) and more precision with a high volume of samples and under conditions of homogeneity and heterogeneity [8]. Therefore, in this study, rates of underreporting in sub-groups were calculated according to mixtures.

In order to decrease bias and increase precision, the number of individuals who had attempted DSP was estimated based on different strata, in addition to the general population. Two indices, the Bayesian information criterion and the Akaike information criterion, were used to evaluate the model. The confidence level was calculated using the Monte Carlo method through a probability-based sensitivity analysis for crude and age-adjusted rates and mixtures of truncated count data. Crude suicide rates were calculated using the average population of the city based on 2011 census data and registry office information [16]. Directly standardized rates were calculated using the World Health Organization (WHO) world standard population [17]. Additionally, the degree to which available data completely identified the total number of individuals who had attempted suicide was calculated using a mixture of methods and by dividing the observed values (direct method) by the estimated values.

Stata version 13.1 (StataCorp., College Station, TX, USA) and computer-assisted mixture model analysis in the Capture-Recapture (http://www.pitt.edu/~yuc2/cr/main.htm_) count data software were used to analyze data and estimate the rate of underreporting, respectively [18].

### Ethical considerations

The present study was conducted according to the Helsinki Declaration. Those who had attempted DSP were interviewed personally by a group of Imam Khomeini Hospital psychologists. The study and its related methods were approved by the Ethics Committee of Kerman University of Medical Sciences (IR. KMU. REC. 2015. 440).

## RESULTS

A total of 1,790 individuals attempted DSP (ICD-10 codes: X60-X69), 1,023 (57.2%) of whom were female and 767 (42.8%) of whom were male. Among these individuals, 64 subjects (20 women, 44 males) died of severe poisoning. Death from severe poisoning among men was found to be significant (p < 0.001). The mean age for females was 25.2± 11.0 years and the mean age for males was 24.4± 8.0 years. There was no statistically significant difference in age between sex (p = 0.1).

The most commonly used poisoning substances were, in decreasing order, medications (ICD-10 codes: X60-X64), agricultural toxins (ICD-10 codes: X68, X69), and drugs and alcohol (ICD-10 codes: X65, X62), which were used by 1,315 individuals (76.2%), 304 individuals (17.6%), and 44 individuals (2.5%), respectively. The most commonly used medications were tramadol (n= 235; 17.8%), psychotropic drugs (n= 129; 9.8%), and methadone (n= 54; 4.09%). Among the failed attempted suicides, 657 (43.8%) individuals had a history of SA. The highest number of attempted suicides by an individual was 15 (Table 1).

Regardless of heterogeneity (strata), the number of attempted suicides in western Iran was estimated by the Zelterman, Chao, and mixture methods as 3,689, 3,425 and 3,238 cases, respectively. The mixture method showed the lowest root MSE (Table 2).

With the mixture estimation, no significant differences were observed among sub-groups after considering heterogeneity. A calculation of the strata-based population using the mixture method provided better estimates than the other truncated count models (Table 3).

The crude rates of the number of attempted suicides by DSP estimated by the direct and mixture methods were 167.5 per 100,000 persons and 331.7 per 100,000 persons, respectively. After adjusting by the WHO world standard population, the rates of the number of attempted suicides by DSP estimated by the direct and mixture methods reached 129.8 per 100,000 persons and 253.1 per 100,000 persons, respectively (Table 4).

**Figure 1.**
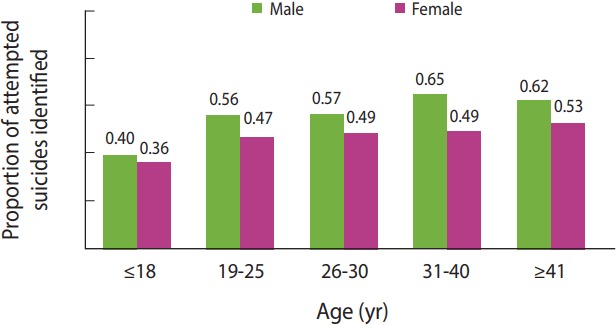
Estimated proportion of attempted suicides by sex and age group.

The degree to which available data completely identified the total number of individuals who had attempted suicide was determined by various estimators and ranged from 46.6-53.2% of the actual number of individuals. The rate of underreporting decreased as the potency and intensity of poisoning agents increased. The maximum rates of underreporting were related to the use of oil and detergents (ICD-10 code: X66), medications (ICD-10 codes: X60-X64), and agricultural toxins (ICD-10 codes: X68, X69) and were estimated as 69.9, 51.2, and 21.5% respectively. As shown in Figure 1, the degree to which data identified the actual number of individuals who had attempted suicide was higher for males than for females, and the rate of underreporting decreased as age increased.

## DISCUSSION

Studying hidden populations is limited by a lack of reporting and faults in the registration system. In most countries, reported statistics on SA are lower than the actual number, because suicide is recorded as the cause of death only after homicide and accidental death have been rejected as the cause or after a suicide note written by the deceased has been obtained. Therefore, it is possible that some cases of SA are recorded as deaths of undetermined cause due to the difficulty in rejecting other causes of death [19].

After adjusting by age based on the WHO world standard population, the annual rate of DSP decreased, as the Iranian population was young and younger age groups attempt suicide more frequently. In comparison to other studies performed in other parts of Iran, the present study showed higher annual rates of SA by DSP [20,21]. However, in comparison to similar studies conducted in other countries, the rates of SA by DSP did not substantially differ, even after consideration of underreporting. For example, the rate of SA by DSP has been estimated as 315 per 100,000 persons (330 per 100,000 males, 299 per 100,000 females) in Sri Lanka [22] and 133.75 per 100,000 persons in the UK [23]. In a European multi-center study, rates of SA by medication poisoning were estimated to be 135 per 100,000 males and 188 per 100,000 females in Oxford, 211 per 100,000 males and 188 per 100,000 females in Helsinki, and, on average, 172 per 100,000 for both sexes in Stockholm [24]. The rate of DSP for those who were referred to hospitals in the US was found to be 76 per 100,000 persons (66 per 100,000 males and 101 per 100,000 females) [25].

In Asia, rates of SA are higher among males than females, while the opposite is true in Europe [26]. The rates of DSP in Asian countries are nearly 20-30% greater than the rates in European and Western countries [25,27] for reasons including availability of poisons [28], urbanization [29], and socioeconomic differences [30]. In rural areas and Asian countries, chemicals and agricultural toxins are the most commonly used substances for DSP, and in urban areas and most European countries, more than 70% of poisoning substances are accounted for by medications, in particular psychotropic drugs [24].

In this study, the highest rate of SA was observed among individuals aged 19-25 years, which agrees with the findings of other studies performed in this field. From the ages of 19-25 years, rates of SA have been found to be 2-4 times higher than the rates of SA in other age groups [22,25]. Since rates of SA are generally high in this age group and more than 20% of the Iranian population falls into this age category, it is necessary for authorities and health care providers to collaborate more vigorously in order to find solutions and meet the needs of this group.

Despite more than 90% of the non-deliberate and deliberate poisoning cases in Kermanshah being referred to the Imam Khomeini Treatment Center, all estimates indicated that only approximately 50% of all individuals who had attempted suicide were treated at this center. This finding concurs with the views of the professionals and psychologists in the poisoning ward who believed that half of these individuals did not receive treatment or counseling and psychological services because they had denied their SA or were not referred to treatment centers [30].

The rate of underreporting is higher among females than males, which is most likely due to the stronger taboo of suicide among females. As the virulence and fatality of the poisoning agents became more severe, the rate of underreporting decreased, such that the rates of denial and underreporting were less for agricultural toxins than for oil and detergents due to their greater toxicity. Similar studies have shown that more potently toxic substances increased the likelihood of being taken to the hospital and decreased the likelihood of denial and non-registration of the SA [29,30].

The rate of SA underreporting decreased with an increase in age, with the highest rate of underreporting observed in those 18 years old or younger. Generally, rates of denial and underreporting of SA are higher among individuals who attempt suicide for the first time than those who have a history of SA. One reason that rates of underreporting decrease as age increases is that younger people are more likely to use a greater amount of more potent substances for poisoning.

The Chao and Zelterman estimators that were derived under homogeneity when the number of components was low (k< 3) performed reliably, which is consistent with similar studies [8]. The proposed estimator (the mixture estimator) is an excellent estimator with the smallest bias value. This study showed that the mixture estimator had a small bias value when the data had heterogeneity. For data sets under homogeneity and heterogeneity, the mixture of the truncated Poisson models provided the best population estimates. The Chao and Zelterman estimators provided a lower and upper bound value, respectively, and the mixture of truncated count models provided a value between those of the Chao and Zelterman estimators.

### Strengths and weaknesses of the study

The strengths of this study include using several information sources to determine the degree to which data identified all attempted suicides, employing clinical psychologists to perform interviews, and using precise systems to record information about individuals who attempted DSP. However, like other similar studies in this field, weaknesses of this study include a lack of cooperation from some individuals who had attempted suicide, limited and difficult access to information from different centers, a lack of precise information on the type of drugs and substances used for suicide, and limited statistical methods for estimating the rates of underreporting. The main limitations in truncated count models are the small values for λ and the heterogeneity of data. Small values for λ cause different methods to give different population estimates. Therefore, it was difficult to determine which method gave the most accurate estimates. In addition, small values for λ result in large confidence intervals and reduce the precision of calculations. Consequently, estimates must be interpreted with greater care when λ values are small in order to avoid overestimating no frequencies.

### Solutions for reducing the rate of underreporting

The underreporting and undercounting of SA occur at different rates around the world. This can be attributed to cases not being reported; faults in the registration system (of mainly developing countries); and political, cultural, and social issues as well as the taboo of SA among individuals and societies at large. Rates of underreporting and undercounting are higher in small societies and in societies where a stronger stigma is placed on SA.

The rates of underreported and unaccounted SA were much higher than the rates of suicides that led to death. The rates of denial and underreporting of SA were much higher among people who had attempted suicide for the first time than those who had a history of SA. Those who have attempted suicide for the first time may conceal their attempts due to fear of social stigma. The rate of denial may decrease if interviews are conducted and individuals are provided assurance in an isolated room. In many countries, including Iran, individuals attempting suicide are not covered to receive treatment under health insurance. Consequently, individuals and their families try to conceal acts of SA in the hope of using insurance coverage. Unfortunately, this may lead to health care staff paying less attention to and spending less time on treating individuals who have attempted suicide. The elimination of the distinction between individuals who have experienced deliberate and non-deliberate poisoning may have a positive effect on reducing the rates of denial and underreporting of SA.

## Figures and Tables

**Table 1.** Observed frequencies of suicide attempts by self-poisoning in western Iran, 2015

No. of suicide attempts by self-poisoning per case (*i*)	0	1	2	3	4	5	6	≥7
No. of cases (*n_i_*)	---	1,069	334	156	78	32	15	30

**Table 2.** Number of attempted suicides by deliberate self-poisoning using the truncated count model

Estimator	Unobserved (n)	Total		RMSE
		n	95% Cl	
Mixture	1,524	3,238	3,127, 3,352	429.4
Chao	1,711	3,425	3,315, 3,535	466.2
Zelterman	1,975	3,689	3,573, 3,805	520.4

CI, confidence interval; RMSE, root mean square error.

**Table 3.** Number of unobserved attempted suicides by deliberate self-poisoning

Stratified variables	Discrete mixture		Observed known population (n)	Mixture estimator (n)	Total (95% confidence interval)
	λ^1^	K^2^			
Sex					3,436 (3,323, 3,539)
Male	2.9	3	723	1,432	
Female	5.0	3	1,003	2,004	
Age (yr)					3, 414 (3,300, 3,531)
≤18	2.2	2	361	1,031	
19-25	3.8	2	797	1,377	
26-30	3.3	2	276	501	
31-40	3.6	2	157	311	
≥41	0.9	1	116	194	
Years of education (yr)					3,336 (3,216,3,452)
0	0.6	1	88	187	
1-6	2.5	2	192	676	
7-9	3.1	2	386	805	
10-13	4.1	2	812	1,363	
≥14	1.0	1	124	305	
Drug abuse					3,324 (3,211,3,427)
Yes	5.0	3	304	478	
No	3.2	2	1,398	2,846	

1 Poisson parameter.

2 The number of mixture components.

**Table 4.** The crude and age-adjusted rates^1^ of attempted suicide by deliberate self-poisoning in 2015

Variable	Direct		Mixture	
	Crude	Age-specific	Crude	Age-specific	
Sex				
Male	146.2	104.1	298.2	205.8
Female	187.2	156.0	363.6	302.8
Age (yr)				
≤18	105.9	42.3	294.7	117.9
19-25	562.9	45.0	1,064.8	85.1
26-30	324.1	25.9	528.9	42.3
31-40	132.7	15.9	213.8	25.6
≥41	41.1	13.1	63.9	20.4
Total	167.5	129.8	331.7	253.1

1 Rate per 100,000 persons.
